# Compartmentalisation of EGFR signalling might potentiate the optimal use of EGFR tyrosine kinase inhibitors in cancer therapeutics

**DOI:** 10.1038/sj.bjc.6603807

**Published:** 2007-05-22

**Authors:** M V Karamouzis, P A Konstantinopoulos, A G Papavassiliou

**Affiliations:** 1 Division of Hematology-Oncology, University of Pittsburgh, Pittsburgh, PA, USA; 2 Department of Biological Chemistry, Medical School, University of Athens, Athens, Greece; 3 Division of Hematology-Oncology, Beth Israel Deaconess Medical Center, Harvard Medical School, Boston, MA, USA

**Sir**,

We have read the recent interesting minireviews by Lo and Hung (2007) and Uramoto and Mitsudomi (2007) regarding epidermal growth factor receptor (EGFR) signalling and we would like to add the following comment to strengthen some issues of this important theme. Ligand-dependent activation of the EGFR induces several signal-transduction pathways as well as trafficking events that relocalise the receptors from the cell surface to intracellular endocytic compartments that provide signal specification. Accordingly, trafficking-dependent alterations in receptor compartmentalisation are crucial regulatory mechanisms for EGFR signalling (Frey *et al*, 2006; Tanos and Pendergast, 2006). In the first step, activated receptors are removed from cell membrane and are internalised into endosomes. In the second step, EGFR may recycle back to the membrane for continued signalling or is targeted for degradation (Orth and McNiven, 2007). The later step is generally appreciated as a signalling-blocking process, although there are data suggesting that internalised receptors might still interact with effector/adaptor proteins and thus activate downstream cascades (Citri and Yarden, 2006). Defective internalisation of ligand-activated EGFR leads to prolonged signalling and has been implicated in malignant transformation (Marmor and Yarden, 2004). This might be partly also related with the ‘short-lived’ observed clinical effect in some cancer patients with the currently used EGFR-TKIs, as it has been suggested that EGFR-TKIs (contrary to monoclonal antibodies) cannot produce long-term EGFR suppression through receptor internalisation and degradation. Moreover, EGFR endocytosis might have important implications in the already tested as well as the future co-administration of chemotherapy and EGFR-TKIs, since it has been recently suggested that the *in vitro* application of EGFR-TKIs before chemotherapeutic agents represses EGFR degradation (Feng *et al*, 2006). Posttranslational modifications are also a prerequisite of EGFR endocytosis. Ras superfamily of GTPases regulate important biologic processes, such as intracellular trafficking. RAB5 has been found to mediate EGFR entry into the early endosome (Barbieri *et al*, 2004; Konstantinopoulos and Papavassiliou, 2007) and RAB11 facilitates EGFR recycling to the plasma membrane (Cullis *et al*, 2002). Conversely, RAB7 is required for the degradation of the ligand-activated EGFR by the lysosome (Ceresa and Bahr, 2006). Thus, modulation of RAB-GTPases could provide an exciting alternative way of EGFR inhibition (Konstantinopoulos *et al*, 2007, in press).
